# Genomic Reconstruction of Carbohydrate Utilization Capacities in Microbial-Mat Derived Consortia

**DOI:** 10.3389/fmicb.2017.01304

**Published:** 2017-07-13

**Authors:** Semen A. Leyn, Yukari Maezato, Margaret F. Romine, Dmitry A. Rodionov

**Affiliations:** ^1^Sanford-Burnham-Prebys Medical Discovery Institute, La Jolla CA, United States; ^2^A. A. Kharkevich Institute for Information Transmission Problems, Russian Academy of Sciences Moscow, Russia; ^3^Biological Sciences Division, Pacific Northwest National Laboratory, Richland WA, United States

**Keywords:** microbial community, carbohydrate utilization, comparative genomics, metabolic reconstruction, transcriptional regulation

## Abstract

Two nearly identical unicyanobacterial consortia (UCC) were previously isolated from benthic microbial mats that occur in a heliothermal saline lake in northern Washington State. Carbohydrates are a primary source of carbon and energy for most heterotrophic bacteria. Since CO_2_ is the only carbon source provided, the cyanobacterium must provide a source of carbon to the heterotrophs. Available genomic sequences for all members of the UCC provide opportunity to investigate the metabolic routes of carbon transfer between autotroph and heterotrophs. Here, we applied a subsystem-based comparative genomics approach to reconstruct carbohydrate utilization pathways and identify glycohydrolytic enzymes, carbohydrate transporters and pathway-specific transcriptional regulators in 17 heterotrophic members of the UCC. The reconstructed metabolic pathways include 800 genes, near a one-fourth of which encode enzymes, transporters and regulators with newly assigned metabolic functions resulting in discovery of novel functional variants of carbohydrate utilization pathways. The *in silico* analysis revealed the utilization capabilities for 40 carbohydrates and their derivatives. Two *Halomonas* species demonstrated the largest number of sugar catabolic pathways. Trehalose, sucrose, maltose, glucose, and beta-glucosides are the most commonly utilized saccharides in this community. Reconstructed regulons for global regulators HexR and CceR include central carbohydrate metabolism genes in the members of Gammaproteobacteria and Alphaproteobacteria, respectively. Genomics analyses were supplemented by experimental characterization of metabolic phenotypes in four isolates derived from the consortia. Measurements of isolate growth on the defined medium supplied with individual carbohydrates confirmed most of the predicted catabolic phenotypes. Not all consortia members use carbohydrates and only a few use complex polysaccharides suggesting a hierarchical carbon flow from cyanobacteria to each heterotroph. In summary, the genomics-based identification of carbohydrate utilization capabilities provides a basis for future experimental studies of carbon flow in UCC.

## Introduction

Knowledge of molecular interactions that occur between bacteria in microbial communities is critical for understanding of both environmental and human-associated ecological niche formation. Because natural microbial communities are typically complex in terms of species diversity and function, simplified models are necessary to study the basis of their behavior. Recently two nearly identical unicyanobacterial consortia (UCC) were derived from a photosynthetic microbial mat from Hot Lake, Washington (Cole et al., 2014) and their member genomes assembled from metagenome and isolate DNA sequence (Nelson et al., 2015). Since the single cyanobacterial member of each consortium is also the only autotroph, their heterotrophic cohorts depend on the cyanobactium for organic carbon when CO_2_ is the only external source of carbon provided. Previous experimental data suggest the presence of numerous metabolic interactions between the heterotrophs and the cyanobacterium, which makes these consortia excellent models for defining the metabolic interactions potential in the community (Cole, 1982; Carpenter and Foster, 2002; Seymour et al., 2010; Beliaev et al., 2014).

Cyanobacteria excrete numerous organic compounds including fermentation products (Stal and Moezelaar, 1997), osmolytes (Hagemann, 2011), polysaccharides, proteins, nucleic acids, and lipids (Decho, 1990; Chiovitti et al., 2003; Flemming and Wingender, 2010). The UCC cyanobacteria form sheaths made up of exopolysaccharides (EPSs), high-molecular-mass heteropolymers composed of various sugars and their derivatives. Electron microscopy of the UCC show that heterotrophic bacteria are attached to these sheaths, which suggests that their constituents could be used as carbon source for the heterotrophic members (Cole et al., 2014). Cyanobacterial EPSs are characterized by their complex structure with high diversity of monosaccharides found as their building blocks. Up to 75% of EPSs are heteropolysaccharides composed of least six different types of monosaccharides (Pereira et al., 2009). The most common carbohydrates found in EPSs of Cyanobacteria are glucose, galactose, mannose, fructose, fucose, rhamnose, xylose, arabinose, as well as glucuronic and galacturonic acids (Pereira et al., 2009). The composition of EPSs produced by the UCC cyanobacteria is currently unknown, however, the previous metabolic analysis of UCC composition identified an abundance of putative osmolytes such as glycerol, gluconate, glucosylglycerol, glucosylglycerate, sucrose, and trehalose (Cole et al., 2014).

A highly diverse array of metabolic pathways for utilization of carbohydrates has been previously described for heterotrophic bacteria (Yang et al., 2006; Gu et al., 2010; Leyn et al., 2012; Rodionova et al., 2012, 2013a,b; Zhang et al., 2012). The sugar utilization networks in bacteria are represented by a large number of species-to-species variations in carbohydrate hydrolases, uptake transporters, transcriptional regulators and enzymes catalyzing the catabolism of monosaccharides. A subsystems-based comparative genomics approach allows us to substantially enhance the accuracy of genomic annotations, to infer functions of previously unknown gene families and to describe metabolic pathways and associated transcriptional networks in several diverse bacterial taxa (Osterman and Overbeek, 2003; Overbeek et al., 2005; Rodionov, 2007). The subsystems approach was highly efficient for prediction of novel sugar catabolic pathways that are often comprised of co-localized and co-regulated genes. The applicability and efficacy of this *in silico* approach was shown by our previous reconstructions of sugar utilization networks in *Bacteroides*, *Shewanella*, and *Thermotoga* genera (Rodionov et al., 2010, 2013; Ravcheev et al., 2013) and by similar works of others (Warda et al., 2016).

Recently, we applied the integrated subsystems-based approach to reconstruct vitamin cofactor biosynthesis pathways and associated transporter capabilities in the 19 organisms that comprise the two model UCC derived from microbial mat from Hot Lake in Washington (Romine et al., 2017) and to predict cofactor exchange among consortial members. In this work, we focused on identification of carbohydrate utilization abilities of the heterotrophic members of these consortia so that we could predict that types of carbohydrates exchanged with the cyanobacterium. Using the bioinformatics approach we systematically mapped peripheral carbohydrate utilization pathways and the central carbohydrate metabolism (CCM) in a group of 17 UCC heterotrophs with sequenced genomes. The reconstructed carbohydrate catabolic network allowed us to annotate a large number of catabolic enzymes, and to infer associated catabolic pathways. In particularly, we identified novel pathway variants such as the predicted pathway for mannoheptulose utilization. In addition, we identified potential transporters and regulators involved in the uptake and sensing of the utilized carbohydrates. The obtained carbohydrate catabolic phenotypes were assessed experimentally using Api50 tests and/or growth of selected UCC isolates on defined media with individual carbohydrates as carbon and energy sources. The combined *in silico* analyses and *in vivo* experiments revealed a large and diverse set of carbohydrate utilization pathways unevenly distributed across the majority of the heterotrophic UCC organisms.

## Materials and Methods

### Bioinformatics Analysis of UCC Genomes

Assembled genomes of 19 UCC members were obtained from the U.S. Department of Energy, Joint Genome Institute (DOE-JGI). The annotated genomic sequences were downloaded from the Integrated Microbial Genome (IMG) expert review database (Chen et al., 2016). In addition, the genomic assemblies can be accessed in the European Nucleotide Archive^1^. UCC is composed of a combination of the species-resolved metagenome bins and isolate genome sequences for organisms that were previously cultivated axenically (Nelson et al., 2015) (**Table 1**). Completeness of genomic content for most of the analyzed metagenomic bins, as previously estimated by presence/absence of 100 conserved single-copy genes, was at least 98%, with the exception of one member, bin09, whose estimated coverage is 88% (Nelson et al., 2015). In addition to the cyanobacterial members, UCC contain 17 heterotrophic members including 10 Alphaproteobacteria, five Gammaproteobacteria, and two species from the *Bacteroidetes* phylum.

**Table 1:** Genomic properties and overview of carbohydrate utilization capabilities of analyzed unicyanobacterial consortia (UCC) organisms.

UCC assembly/isolate genome	Alias	Total genes	GHs^1^	CU genes^2^	CU pathways^3^
**Bacteroidetes**					
*Bacteroidete*s bin01	Bin01	2691	34 (8)	32 (4)	4
*Algoriphagus marincola* HL-49	HL-49	3820	59 (2)	73 (11)	9
**Gammaproteobacteria**					
*Halomonas* sp. HL-48	HL-48	3463	12 (0)	105 (19)	19
*Halomonas* sp. HL-93	HL-93	3928	11 (0)	150 (28)	25
*Aliidiomarina calidilacus* HL-53	HL-53	2602	14 (1)	7 (3)	2
*Marinobacter excellens* HL-55	HL-55	3717	14 (1)	4 (0)	0
*Marinobacter* sp. HL-58	HL-58	3948	28 (1)	45 (3)	6
**Alphaproteobacteria**					
*Roseibaca calidilacus* HL-91	HL-91	3313	22 (1)	46 (13)	8
*Rhodobacteriaceae* bin07	Bin07	3338	16 (0)	8 (6)	1
*Rhodobacteriaceae* bin08	Bin08	3542	34 (0)	164 (41)	18
*Rhodobacteriaceae* bin09	Bin09	3817	27 (0)	49 (15)	11
*Rhodobacteriaceae* bin12	Bin12	3644	15 (0)	7 (0)	0
*Rhodobacteriaceae* bin18	Bin18	3357	24 (0)	75 (19)	12
*Oceanicaulis* bin04	Bin04	2667	9 (0)	0 (0)	0
*Salinivirga fredricksonii* HL-109	HL-109	3832	21 (0)	16 (5)	4
*Erythrobacter* sp. HL-111	HL-111	2862	20 (0)	2 (0)	0
*Porphyrobacter* sp. HL-46	HL-46	3057	14 (0)	15 (4)	4
**Cyanobacteria**					
*Phormidium* OSCR	OSCR	4426	30 (0)	n/a	n/a
*Phormidesmis priestleyi* ANA	ANA	4911	37 (0)	n/a	n/a


**FIGURE 1:**
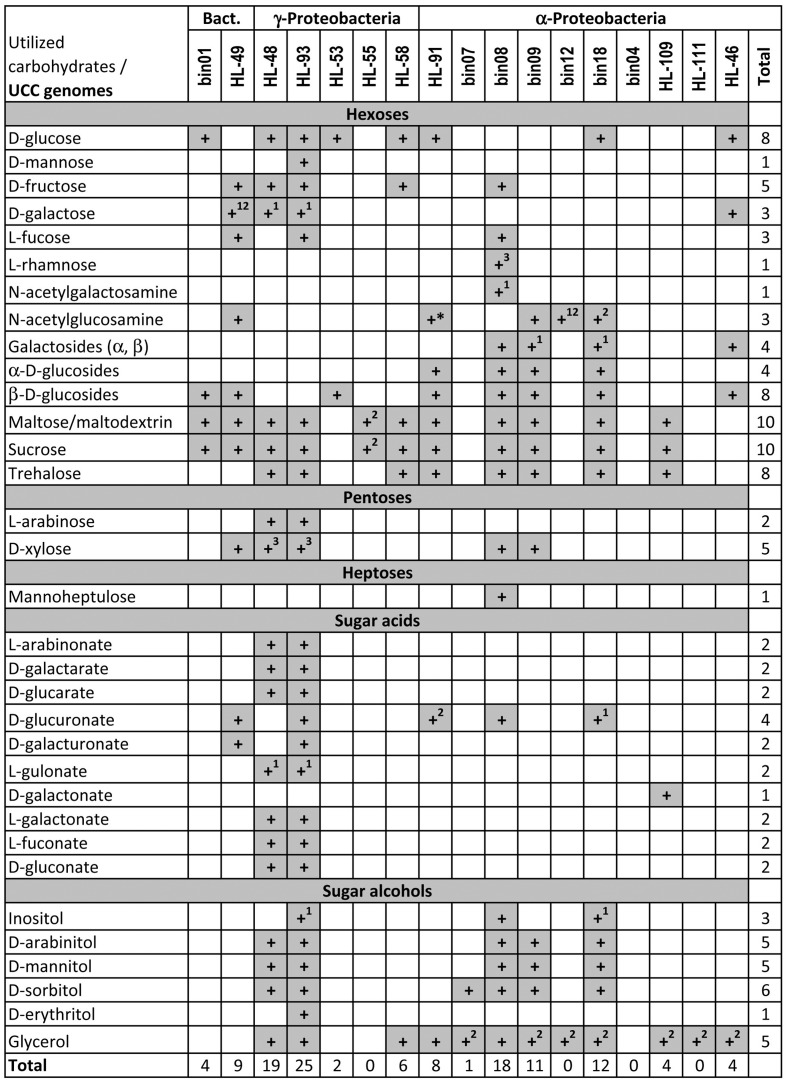
Predicted carbohydrates utilization capability of heterotrophic unicyanobacterial consortia (UCC) organisms. Aliases for analyzed UCC genomes are described in **Table 1**. The ability of UCC species to grow on a panel of sugar substrates was predicted based on the presence of the respective reconstructed pathways and carbohydrate-specific transporters in their genomes. Superscript numbers indicate that: (1) the reconstructed pathway contains a missing enzyme; (2) the catabolic pathway is present but carbohydrate-specific transporter is missing; (3) the catabolic pathway includes uncharacterized enzymes with unclear biochemistry. Asterisk indicates the predicted ability to utilize *N-*acetylmuramic acid (the ether of lactic acid and *N*-acetylglucosamine), which is based on the presence of MurQ etherase and the complete *N*-acetylglucosamine catabolic pathway.

*^1^Number of predicted glycosyl hydrolases (GHs) per genome. Number of predicted extracellular GHs is given in parenthesis. The details on prediction of GHs and bioinformatics assignment of their cellular localization are given in Supplementary Table S1. ^2^Number of genes involved in the reconstructed carbohydrate utilization (CU) pathways including enzymes, uptake transporters and transcriptional regulators. Number of genes with newly assigned functions is given in parenthesis. The details of all functional assignments are provided in Supplementary Table S2. ^3^Number of reconstructed carbohydrate utilization pathways reflects an estimate of a number of different carbohydrates utilized through the reconstructed pathways. Incomplete CU pathways with missing carbohydrate transporters are not counted. Detailed distribution of CU pathways is given in **Figure 1**.*

Glycoside hydrolases (GHs) were predicted by analyzing the deduced proteome sequence from all 19 UCC organisms on the dbCAN server (Yin et al., 2012). Cellular localizations of GHs were predicted as previously described (Romine, 2011). Briefly, genomes were assessed for the presence of secretion systems to identify those capable of secreting proteins and then deduced GH sequences analyzed with the following web-tools: SignalP with sensitive parameters (0.5 SignalP-noTM/0.42 SignalP-TM) (Petersen et al., 2011); LipoP (Juncker et al., 2003); TatP (Bendtsen et al., 2005b); SecretomeP (Bendtsen et al., 2005a); TMHMM (Krogh et al., 2001); PRED-TMBB2 (Tsirigos et al., 2016); PSORTb (Yu et al., 2010); and SOSUIGramN (Imai et al., 2008). Predictions of localization were additionally improved based on the presence of location-informative domains and the assumption that orthologous GHs should have same subcellular localization. Identification of orthologs in closely related genomes was performed using IMG. Functional annotations of predicted GHs were manually curated with input from the UniProt database (Boutet et al., 2016) and the RAST annotation server (Overbeek et al., 2014).

### Genomic Reconstruction of Metabolic Pathways and Regulons

The UCC genomes were previously annotated via the following two pipelines: (i) the DOE-JGI Microbial Genome Annotation Pipeline (Huntemann et al., 2015), and (ii) the RAST server (Overbeek et al., 2014). First, we obtained the set of genes that are potentially involved in the carbohydrate metabolism in UCC genomes by filtering the RAST-based gene annotations and subsystem assignments, and by adding the predicted sets of functionally annotated GHs. The initial gene set was further expanded by potential carbohydrate metabolism genes according to their KEGG Orthology (KO) annotations (Kanehisa et al., 2016). Finally, we added genes from specific protein families associated with carbohydrate metabolism in the Pfam database according to their Gene Ontology terms (Finn et al., 2016). The expanded set of genes potentially involved in carbohydrate metabolism was further analyzed using manual inspection and the genome context techniques. We used the following three genome context techniques to functionally link a set of genes to a single pathway: (i) clustering of genes on the chromosome (operons), (ii) co-regulation of genes by a common regulator (regulons), and (iii) co-occurrence of genes in a set of related genomes (Overbeek et al., 2007; Rodionov, 2007; Haft, 2015).

Reconstruction of carbohydrate utilization pathways in 17 heterotrophic UCC members was performed using the subsystem-based comparative genomics approach combined with genomic reconstruction of carbohydrate-specific transcription factor (TF) regulons and identification of candidate carbohydrate-specific transporters as previously described (Rodionov et al., 2010, 2013; Ravcheev et al., 2013). Typical metabolic reconstruction workflow included: (i) analysis of gene neighborhood conservation across closely related microbial genomes using the Gene Ortholog Neighborhood tool in IMG; (ii) BLAST searches for functionally characterized orthologs in SwissProt/UniProt; (iii) reconstruction of local TF regulons to identify additional co-regulated gene loci; (iv) metabolic subsystem analysis for closely related genomes in the SEED database (Overbeek et al., 2014). Many of the initially identified gene candidates whose functional roles were deemed unrelated to carbohydrate utilization (e.g., involved in biosynthetic pathways) were rejected. The refined functional annotations for genes involved in the reconstructed pathways are provided in Supplementary Table S2.

For reconstruction of novel TF regulons, we used the bioinformatics technique based on identification and comparative analysis of candidate TF-binding sites in closely related genomes (Rodionov, 2007) and implemented in the RegPredict software (Novichkov et al., 2010). This approach includes the following steps: (i) search for orthologous groups of the studied TFs in other reference genomes; (ii) selection of conserved orthologous gene loci containing the studied TFs; (iii) prediction of candidate TF binding motifs with palindromic or tandem repeat structures; (iv) construction of positional weight matrices (PWMs) for identified DNA motif and its application for identification of additional sites and regulon members in each TF-containing genome. Scores of candidate sites were calculated as the sum of positional nucleotide weights. The threshold for site scores was defined as the lowest score observed in the training set. The reconstructed regulons included the conserved regulatory interactions in at least two other genomes with TF binding sites above threshold. The CceR and HexR regulons were analyzed using the previously constructed PWMs from the RegPrecise database (Novichkov et al., 2013). Weblogo package (Crooks et al., 2004) was used to build sequence logos for the derived DNA-binding motifs. The reconstructed CceR and HexR regulons are described in Supplementary Table S3. Other identified sugar catabolic regulons and their candidate TF binding sites are provided in Supplementary Table S2.

#### Phenotypic Analysis of Heterotrophic UCC Isolates

An ultimate validation of the genomics-based metabolic reconstructions was attained by experimental testing of growth phenotypes. Four UCC isolates including the *Halomonas* sp. HL-48 and HL-93 strains, *Roseibaca calidilacus* HL-91 and *Marinobacter* sp. HL-58 were tested for their ability to grow on a panel of various carbon sources as a sole carbon and energy source. Cells were grown in Hot Lake Heterotroph (HLH) medium (Cole et al., 2014), containing 10 mM TES pH 8.0, 400 mM MgSO_4_, 80 mM Na_2_SO_4_, 20 mM KCl, 1 mM NaHCO_3_, 5 mM NH_4_Cl, and supplemented with 5 mM of a specific carbohydrate as a sole carbon source. For initial validation of genomic prediction of carbohydrate utilization we used bioMerieux^TM^ Api^TM^50 CH carbohydrate fermentation strips. The Api^TM^50 CH strip contains 49 wells with different carbohydrates in each well and one negative control well (no carbon source). The HLH media for starter cultures was supplemented with 5 mM glycerol (for HL-91) or 5 mM sucrose (for HL-48, HL-93, and HL-58). The mid-log phase grown cells were further washed three times with HLH medium without any carbon source to eliminate carry over of carbon substrates from starter media prior to inoculation and washed cells were used as inoculums in Api50 CH strip. Utilization of carbon sources were indicated by the growth on the well. For each strain, two independent repetitions were performed. The incubation time for Api50 CH strip measurements was 3 days (for HL-48, HL-93, and HL-58) and 7 days (for HL-91). Growth phenotype of Api50 CH strip results were further validated by growth measurements using selected carbohydrates. An optical density (OD_600_) was measured to monitor cell growth during 60–100 h using a plate reader instrument Norden Lab Professional-Bioscreen. 250 μL culture volumes in the 100 well Bioscreen plate, and each growth experiments were performed in 10 replicates.

## Results and Discussion

### Glycoside Hydrolases

To estimate sugar degradation capabilities of UCC members, we identified sets of carbohydrate active glycosyl hydrolases (GHs) that are involved in breakdown of oligosaccharides (and polysaccharides) into monosaccharides. Overall, 441 proteins containing at least one GH domain were found unevenly distributed in the studied UCC genomes (**Table 1**). The majority of the identified GHs have a predicted cellular localization either in the cytoplasm (for 217 GHs) or in the periplasm (for 178 GHs), with an additional 29 GHs found in the inner membrane (Supplementary Table S1).

The type two generalized protein secretion system was found in only four heterotrophs; *Aliidiomarina calidilacus* HL-53, *Marinobacter* sp. HL-58, *Marinobacter excellens* HL-55, and *Oceanicaulis* bin04 but are predicted to only secrete a single GH per genome, except bin04 which has no predicted extracellular GH (Supplementary Table S1). HL-53 also encodes a single outer membrane GH. The type IX protein secretion system was found in *Bacteroidetes* bin01 and *Algoriphagus marincola* HL-49 and is predicted to be responsible for secreting eight and two GHs, respectively. *R. calidilacus* HL-91 and *Rhodobacteriaceae* bin12 and bin18 encode a single autotransporter that secretes an orthologous cellulase. Collectively these results suggest that at least eight heterotrophs are able to degrade polysaccharides and that the remaining heterotrophs may rely on them for production of mono- and disaccharides that can be transported into the cell for further degradation or that they utilize other forms of carbon to support their carbon and energy needs.

Using similarity searches against the Uniprot database and metabolic reconstructions via the comparative genomics techniques (see below) we analyzed potential function of 374 GHs identified in the heterotrophic UCC organisms. As result, we tentatively assigned substrate specificity and metabolic pathway to 338 GHs (Supplementary Table S1). Of these, 125 of the GH enzymes are membrane-bound or periplasmic transglycosylases that are involved in peptidoglycan metabolism. An additional 53 GHs are putatively involved in biosynthesis of trehalose, maltose, or glycogen. The remaining 160 GHs with assigned functional roles are potentially involved in carbohydrate utilization pathways, at that 112 and 29 of them are potentially located in the cytoplasm and the periplasm, respectively. The remaining functionally annotated GHs with catabolic functions are distributed between the periplasm, the inner and outer membranes and the extracellular milieu. Among 12 extracellular GHs there are six β- and five α-glucosidases involved the glucan and maltodextrin utilization, as well as a probable chitinase. Half of these secreted GHs were from *Bacteroidetes* bin01, suggesting it is an important UCC member contributing to initial breakdown of polysaccharides.

### Peripheral Carbohydrate Utilization

We applied subsystem-based comparative genomics approach to reconstruct peripheral carbohydrate utilization pathways in the 17 heterotrophic UCC members. Our analysis revealed highly diverse capabilities of UCC organisms to utilize carbohydrates and their derivatives (**Figure 1**). Overall, we identified pathways for utilization of six hexoses (glucose, galactose, fructose, mannose, fucose, rhamnose), two amino sugars (*N*-acetylgalactosamine and *N*-acetylglucosamine), two pentoses (arabinose and xylose), 10 sugar acids and diacids (see below) and six sugar alcohols including inositol, arabinitol, mannitol, sorbitol, erythritol, and glycerol. In addition to monosaccharides, we reconstructed catabolic pathways for several oligosaccharides including α- and β-glucosides, α- and β-galactosides, maltose, sucrose, and trehalose. Finally, we predicted a novel putative pathway for utilization of mannoheptulose (a heptose).

Most heterotrophic UCC members were predicted to catabolize at least one carbohydrate (**Table 1**). *M. excellens* HL-55, *Erythrobacter* sp. HL-111, *Rhodobacteriaceae* bin12, and *Oceanicaulis* bin04 lack any complete carbohydrate utilization pathway although some of them possess some catabolic genes in the absence of predicted carbohydrate-specific transporters. UCC members with a limited number of carbohydrate utilization pathways include *A. calidilacus* HL-53 (predicted to utilize glucose and β-glucosides) and *Rhodobacteriaceae* bin07 (only has sorbitol utilization pathway). In contrast, two *Halomonas* species (HL-48 and HL-93) and *Rhodobacteriaceae* bin08 have the largest numbers of identified carbohydrate utilization genes and pathways. For instance, HL-93 has the predicted capabilities to utilize 25 carbohydrates and their derivatives, whereas HL-48 and bin08 have 18–19 individual pathways.

The peripheral carbohydrate utilization pathways include between eight proteins in HL-111 to up to 150 proteins in HL-93 (**Table 1**). The complete list of 798 proteins involved in the reconstructed pathways across 16 organisms along with their deduced functional annotations is provided in Supplementary Table S2. Nearly half of these proteins constitute metabolic enzymes including nearly 100 of GHs with assigned catabolic pathway. The set of 285 annotated proteins are components of almost 100 carbohydrate transport systems. The obtained metabolic reconstruction includes 88 DNA-binding TFs that presumably control the reconstructed carbohydrate catabolic pathway genes. Using the metabolic reconstruction approach, we predicted specific functional assignments for 171 proteins, whose functions were previously unknown or annotated only at the level of general class (**Table 1** and Supplementary Table S2). Below, we describe the key novel aspects of the reconstructed catabolic pathways in UCC organisms in more details.

#### L-Arabinose and L-Arabinonate Utilization

**FIGURE 2:**
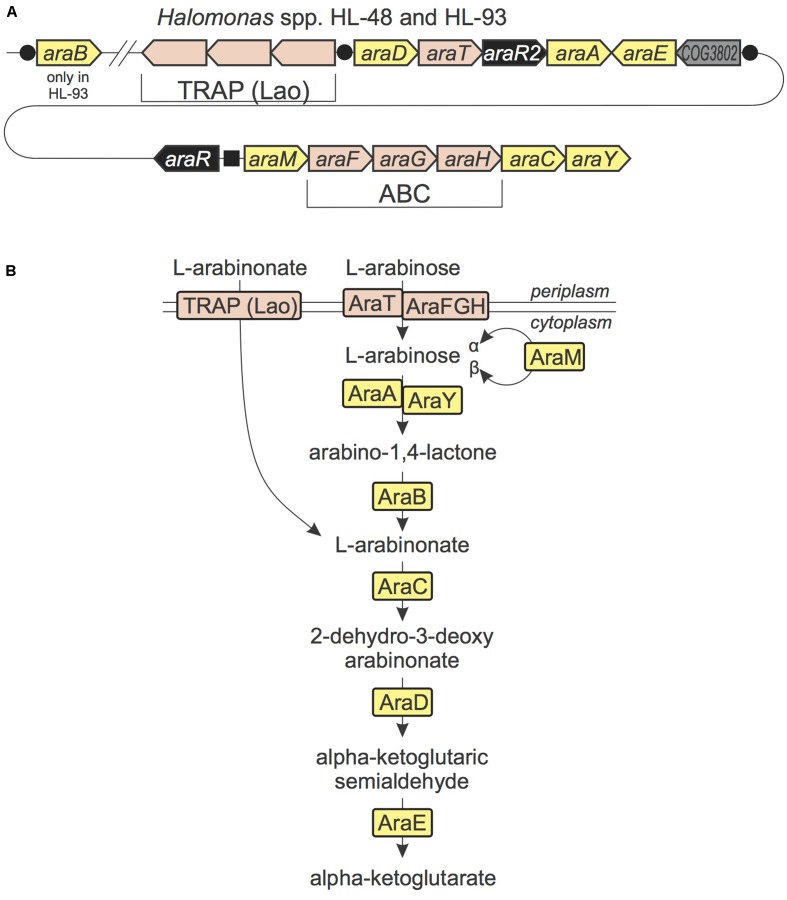
L-Arabinose and L-arabinonate utilization in *Halomonas* species. **(A)** The conserved locus in HL-48 and HL-93 genomes containing genes from L-arabinose and L-arabinonate utilization pathways. Genes are colored by their general functional roles: enzymes (yellow), transcription factors (black), transporters (pink), uncharacterized genes (gray). Candidate TF binding sites are shown as black circles (for AraR2) and a black square (for AraR). **(B)** Scheme of L-arabinose and L-arabinonate utilization pathways in *Halomonas* spp. Enzymes are colored by matching colors as in **(A)**.

In both studied *Halomonas* species we found a new gene locus potentially involved in the utilization of L-arabinose and L-arabinonate (**Figure 2**). It encodes proteins that are orthologous to (i) the ABC-type arabinose uptake transporter system AraFHG and (ii) the AraA, AraC, and AraE enzymes from the oxidative arabinose degradation pathway in *Azospirillum brasiliense* (Watanabe et al., 2006a,b). Based on genome context and distant homology analysis we have identified candidates for the missing 2-keto-3-deoxy-L-arabonate dehydratase (AraD) and a second isozyme of arabinose-1-dehydrogenase (AraY). A member of aldose 1-epimerase family encoded in the *ara* gene cluster was previously assigned the functional role L-arabinose mutarotase (AraM), which interconverts alpha and beta anomers of L-arabinose. The *ara* gene locus in *Halomonas* encodes two novel TFs from the LysR and GntR families (named AraR and AraR2, respectively). Reconstruction of their cognate regulons using these and other *Halomonas* genomes has revealed two different DNA motifs (Supplementary Table S2). AraR presumably controls the divergently transcribed *araMFGHCY* and *araR* genes, whereas AraR2 is predicted to co-regulate the *araD-araT-araR2-araA* operon, the *araE* gene and several other genes encoding a novel transporter from the tripartite ATP-independent periplasmic (TRAP) transporter family and a hypothetical lactonase, which was assigned the missing arabinolactonase function (named AraB). Nearly all known TRAP-family transporters have specificities to organic acids (Vetting et al., 2015), suggesting the novel AraR2-regulated transporter is specific to L-arabinonate, an intermediate of the L-arabinose catabolism. In summary, the *ara* gene locus represents interconnection of two regulatory systems controlling a shared catabolic pathway for utilization of L-arabinose and L-arabinonate.

#### L-Fucose, L-Fuconate, and L-Galactonate Utilization

**FIGURE 3:**
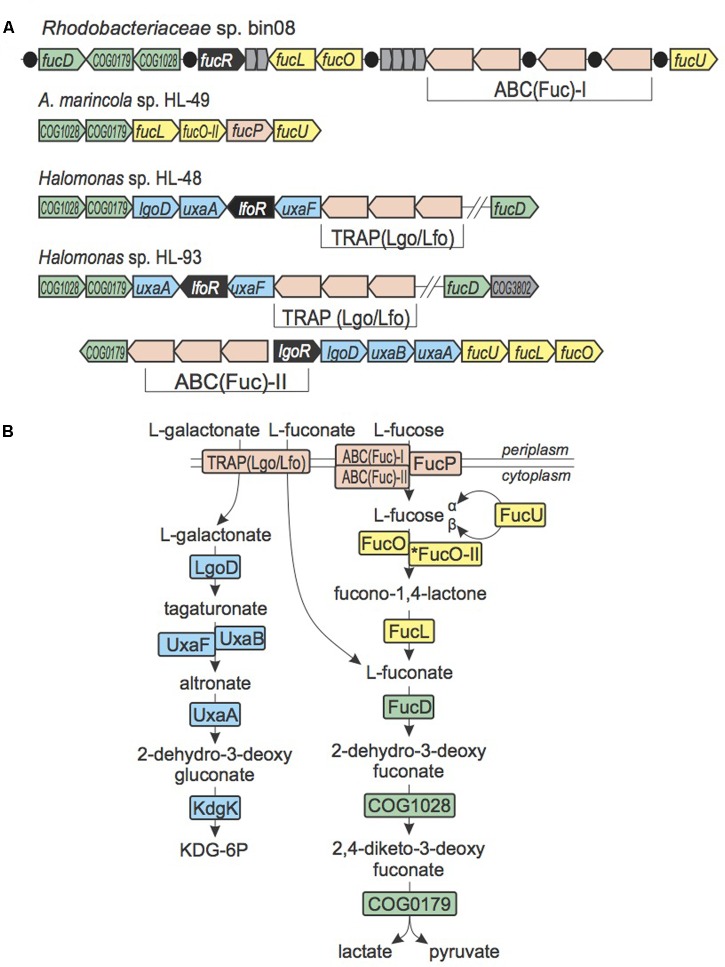
L-Fucose, L-fuconate, and L-galactonate utilization in UCC bacteria. **(A)** Loci containing L-fucose, L-fuconate, and L-galactonate utilization genes. Genes are colored by their general functional roles: transcription factors (black), transporters (pink), uncharacterized genes (gray), enzymes from L-galactonate utilization pathway (blue), enzymes from the L-fucose utilization pathway (yellow), enzymes from the L-fuconate utilization pathway (green). Predicted DNA binding sites of FucR are shown as black circles. **(B)** Scheme of L-fucose, L-fuconate, and L-galactonate utilization pathways. Enzymes are colored by matching colors as in **(A)**.

The oxidative pathway for utilization of L-fucose, where L-fuconate is an intermediate, was shown in *Xanthomonas campestris* (Yew et al., 2006). We observed loci containing genes from this pathway in both *Halomonas* species (HL-48 and HL-93), *A. marincola* HL-49 and *Rhodobacteriaceae* bin08 (**Figure 3**). HL-48 possesses genes encoding the last two steps of this pathway, COG0179 and COG1028. However, the absence of genes for the first two steps of the fucose pathway suggests that HL-48 can only utilize the L-fuconate intermediate. In contrast, HL-93 and bin08 have the complete L-fucose utilization pathway, whereas in HL-49 L-fuconate dehydratase FucD is missing and L-fucose dehydrogenase is substituted with a novel non-orthologous dehydrogenase (named FucO^II^). The four UCC genomes have different transporters encoded in the *fuc* loci. HL-49 has a gene encoding an ortholog of fucose permease FucP from *X. campestris*. HL-93 and bin08 have two non-orthologous ABC systems that we predict to be involved in uptake of L-fucose.

The L-fucose/L-fuconate utilization gene loci in both *Halomonas* species contain the *lgoD* gene encoding L-galactonate-5-dehydrogenase, which is the signature gene of L-galactonate utilization (Kuivanen and Richard, 2014), the *uxaA* and *uxaB* (or *uxaF*) genes involved in the downstream steps of L-galactonate catabolism, as well as a novel TRAP-family transporter operon (**Figure 3**). A similar L-galactonate catabolic gene locus in *Chromohalobacter salexigens* (Csal_1738-1731) encodes the same set of catabolic enzymes and a non-orthologous TRAP transporter, which was previously characterized to have a dual specificity toward L-fuconate and L-galactonate (Vetting et al., 2015). Based on these observations, we propose that the novel TRAP system encoded within the L-fucose/L-fuconate/L-galactonate gene loci in *Halomonas* species is involved in the utilization of both L-fuconate and L-galactonate and thus was named Lgo/Lfo. This example introduces an interesting case of chromosomal co-localization (and, likely, co-regulation) of genes that are involved in the shared carbohydrate utilization pathways.

#### Utilization of Hexuronic Acids, Hexose Diacids, and L-Gulonate

**FIGURE 4:**
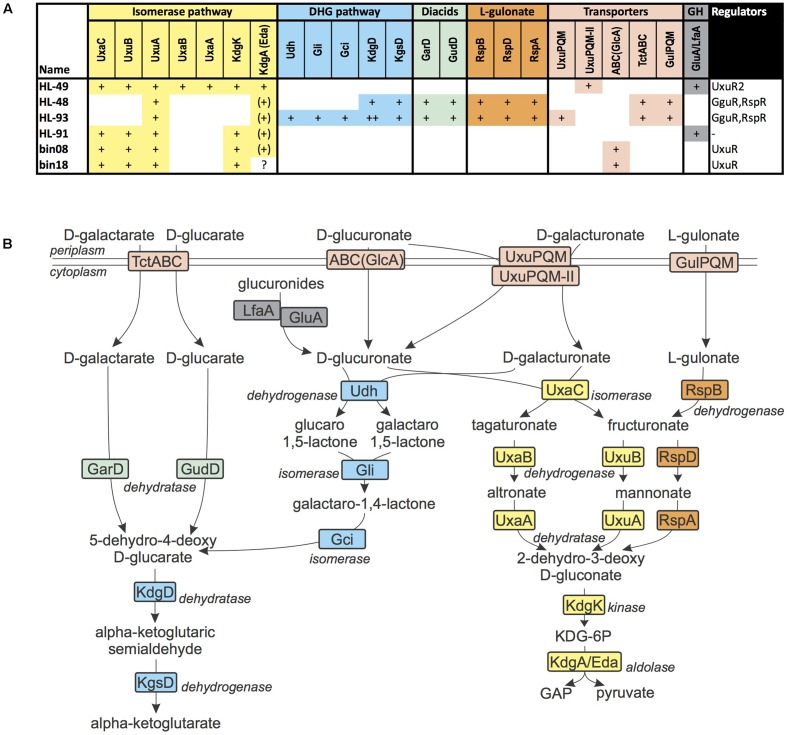
D-Glucuronate, D-galacturonate, D-glucarate, D-galactarate, and L-gulonate utilization in UCC bacteria. **(A)** Distribution of enzymes from the sugar acid utilization pathways in UCC genomes. Presence of enzymes is marked by “+” for each gene with a given function. Each pathway colored by distinct color. Enzymes from central carbohydrate metabolism (CCM) are marked by “(+).” Missing enzymes are marked by “?” **(B)** Scheme of sugar acid utilization pathways in UCC genomes. Enzymes are colored by matching colors as in **(A)**.

D-Galacturonate and D-glucuronate are hexuronic acids that are commonly found in pectins, proteoglycans and glucuronans. D-Galactarate and D-glucarate are the ring opened hexose diacids (or aldaric acids) that serve as a growth substrate to many microorganisms. Both *Halomonas* species (HL-48 and HL-93) contain a gene cluster encoding enzymes involved in the galactarate/glucarate utilization, as well as a novel predicted transporter from the tripartite tricarboxylate transporter (TTT) family (named TctABC) and a novel GntR-family TF (termed GguR). The TctABC transporter is predicted to be involved in galactarate/glucarate uptake. The reconstructed GguR regulon in both *Halomonas* genomes includes the galactarate/glucarate utilization operon, whereas HL-93 has an additional GguR-regulated operon, which encodes the glucoronate/galacturonate utilization enzymes Udh, Gli, and Gci, as well as an ortholog of the known hexuronate transporter UxuPQM. We concluded that HL-93 (but not HL-48) has an additional capability to utilize galacturonate and glucoronate using the pathway partially shared with the galactarate/glucarate pathway (**Figure 4**).

The *R. calidilacus* HL-91 and *Rhodobacteriaceae* bin08 and bin18 genomes encode a different variant of the glucuronate utilization pathway, which starts from the UxaC isomerase and continues through 2-dehydro-3-deoxygluconate (KDG) and its phosphorylated derivative, KDG-6P. The corresponding glucoronate utilization gene loci include a novel ABC-family transporter operon, which was predicted to be co-regulated with the glucuronate utilization genes by a novel GntR-family regulator (UxuR). However, the corresponding transporter and regulator are missing in HL-91, thus the mechanism of glucuronate uptake in yet unknown in this organism. We speculate that it can take up glucuronides that are hydrolyzed in the cytoplasm by the LfaA glucosidase, thus providing glucoronate to feed the catabolic pathway.

*Algoriphagus marincola* HL-49 has two separate loci with galacturonate and glucuronate utilization genes. Both of these gene loci are controlled by a novel LacI-family TF (UxuR2) and encode a novel or TRAP-family transporter (UxuPQM^II^) and an uncharacterized glycosyl hydrolase from the GH109 family. UxuPQM^II^ is distantly related to the previously characterized UxuPQM transporters in various Proteobacteria (Vetting et al., 2015), however, it belongs to a distinct orthologous group of TRAP transporters that are mostly present in the *Bacteroidetes* phylum. We propose that UxuPQM^II^ also has the dual specificity for both hexuronic acids it is co-regulated with both galacturonate and glucuronate utilization genes in HL-49.

In both studied *Halomonas* genomes, we identified a conserved locus encoding proteins homologous to catabolic enzymes, a TRAP-family transporter and a GntR-family regulator that were previously characterized as a part of the L-gulonate utilization pathway in *C. salexigens* (Wichelecki et al., 2014). Thus, we predict that HL-48 and HL-93 are able to utilize L-gulonate.

#### D-Galactose, D-Galactosides, and D-Galactonate Utilization

**FIGURE 5:**
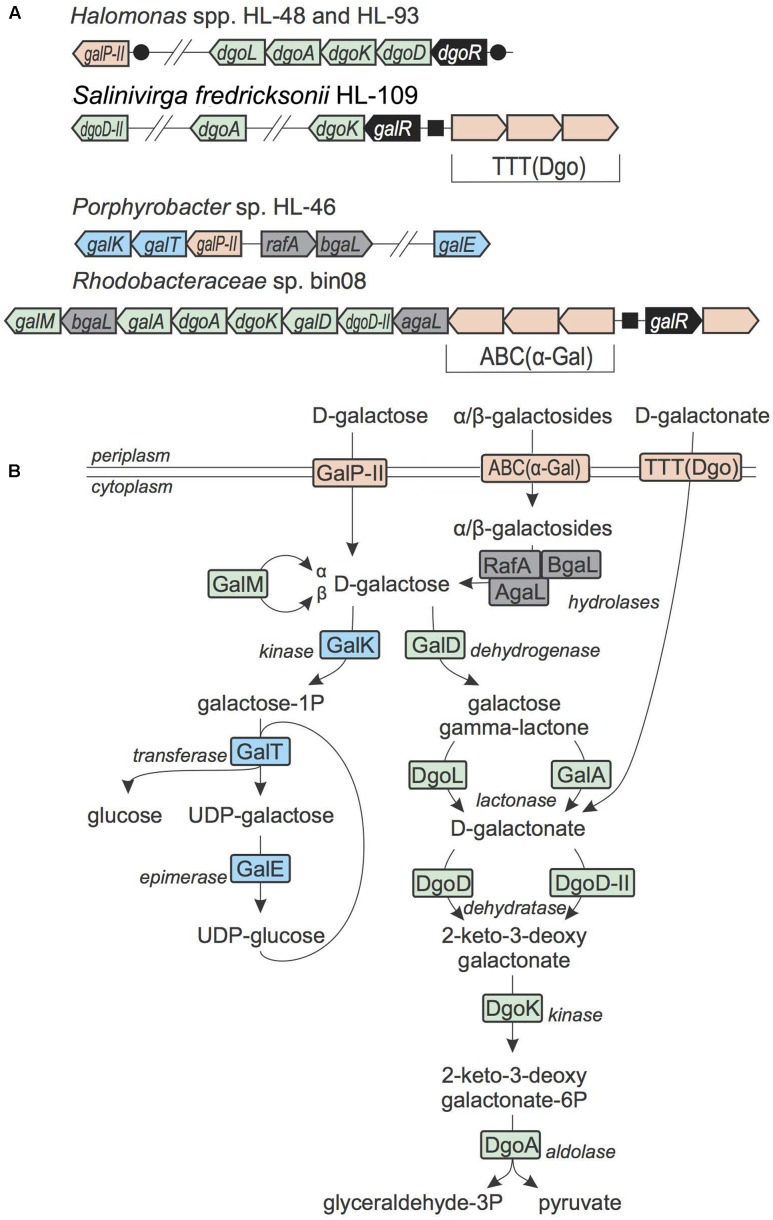
D-Galactose, galactoides, and D-galactonate utilization in UCC bacteria. **(A)** Loci containing D-galactose, galactosides, and D-galactonate utilization genes. Genes are colored by their general functional roles: transcription factors (black), transporters (pink), the Leloir pathway enzymes (blue), the DeLey-Doudoroff pathway enzymes (green), glycosyl hydrolases (gray). Candidate DNA binding sites are shown as circles (for DgoR) and squares (for GalR). **(B)** Scheme of D-galactose, galactosides, and D-galactonate utilization pathways. Enzymes are colored by matching colors as in **(A)**.

In *Escherichia coli* and other Enterobacteria, D-galactose is utilized via the Leloir pathway, which involves galactokinase GalK, galactose-1-phosphate uridylyltransferase GalT and UDP-glucose 4-epimerase GalE (Holden et al., 2003). The *Porphyrobacter* sp. HL-46 and *A. marincola* HL-49 genomes contain the Leloir pathway genes, suggesting there are able to utilize D-galactose (**Figure 5**). The galactose gene locus in HL-46 contains a predicted galactose permease from the SSS family, which is orthologous to the GalP^II^ transporter previously identified in the galactose catabolic gene loci in *Shewanella* spp. (Rodionov et al., 2010). Additionally, the *gal* locus in HL-46 contain two genes encoding cytoplasmic galactosidases, RafA and BgaL, suggesting galactose-containing oligosaccharides may serve as additional inputs to the galactose catabolic pathway. In contrast, the galactose utilization pathway in HL-49 is incomplete with both the GalT uridylyltransferase and a galactose-specific transporter missing.

A different variant of D-galactose catabolic pathway, which is known as the DeLey-Doudoroff pathway, was identified in three *Rhodobacteriaceae* isolates, namely bin08, bin09 and bin18 (**Figure 5**). In this pathway, D-galactose is first oxidized to D-galactonate, which is then converted to pyruvate and GAP through the subsequent action of a dehydratase, a kinase and an aldolase (Wong and Yao, 1994). In addition to the DeLey-Doudoroff pathway enzymes, the galactose utilization gene loci in these three *Rhodobacteriaceae* genomes include genes encoding a cytoplasmic α-galactosidase, an unknown TF from the IclR family and a novel ABC-type transporter. This novel ABC transport system belongs to the Carbohydrate Uptake Transporter-1 (CUT1) family that mostly known to transporter di- and oligo-saccharides, according to the TCDB database (Saier et al., 2014). Thus we propose that this novel transport system is involved in uptake of α-galactosides and that the *Rhodobacteriaceae* spp. are able to utilize as α-galactosides rather than D-galactose.

In *Salinivirga fredricksonii* HL-109, the DeLey-Doudoroff pathway locus is missing the galactose dehydrogenase and lactonase that are required for conversion of D-galactose to D-galactonate. The *dgoK* gene in HL-109 is clustered with genes encoding a novel IclR-family TF (termed GalR) and a novel TTT-family transporter. Known transporters from the TTT family are specific to tricarboxylate and sugar acids (Saier et al., 2014). We predicted that a TTT-family transporter from the incomplete galactose catabolic gene locus is involved in D-galactonate uptake. We also propose that the GalR TF encoded in the same locus senses D-galactonate as an effector. Overall, HL-109 is the only UCC member that is able to utilize this sugar acid.

The DeLey-Doudoroff pathway genes for D-galactonate catabolism are present in *Halomonas* HL-48 and HL-93, as well as in several other reference *Halomonas* genomes. The corresponding *dgo* gene loci contain a hypothetical sugar lactone lactonase from (COG3386, named DgoL), which can serve as a non-orthologous gene displacement for D-galactono-1,4-lactone lactonase GalA. The reconstructed DgoR regulon in the *Halomonas* genomes includes additional candidate co-regulated gene encoding a SSS-family transporter with predicted galactose specificity (GalP^II^). Although orthologs of known D-galactose dehydrogenase (GalD) are missing in *Halomonas* spp., we tentatively assigned them the galactose utilization capability, which is supported by growth phenotype testing (see below). Further similarity searches revealed one possible candidate for the missing GalD reaction in *Halomonas* spp. – a D-xylose dehydrogenase (XylD) from the xylose utilization gene cluster (it has 47% identity with the characterized GalD enzyme from *Rhizobium meliloti*). Thus, we tentatively propose that XylD in *Halomonas* spp. has specificity to multiple substrates including D-galactose and D-xylose.

#### Novel Carbohydrate Utilization Pathway Variants

The reconstructed peripheral pathways in heterotrophic UCC members contain 171 novel genes distinguishing them from those previously described in model species. These include 22 genes encoding novel enzymes with assigned function, 107 genes encoding components of novel sugar transporters and 42 novel sugar-specific transcriptional regulators. Most common are numerous cases of non-orthologous gene displacement, when a novel functional role is encoded by a gene that is not orthologous to any of the previously known genes of the same function. Several predicted non-orthologous enzymes involved in utilization of arabinose (AraB, AraD), fucose (FucO^II^), galactose (DgoD^II^, DgoL), and L-galactonate (UxaF) are described in details above. Other proposed cases of non-orthologous enzymes include a novel *N*-acetylglucosamine kinase (COG1070) in bin09, the putative sorbitol dehydrogenase SorD^II^ in bin07, and the predicted fructokinase MtlZ involved in utilization of arabinitol, sorbitol, and mannitol in both *Halomonas* spp.

Carbohydrate uptake transporters constitute the largest group of newly functionally assigned genes in UCC genomes. Most of these genes encode components of 27 multicomponent transport systems from the ABC, TRAP, and TTT families (Supplementary Table S2). Among 18 novel ABC systems most are predicted to transport hexoses (*N*-acetylglucosamine, *N-*acetylgalactosamine, fucose), oligosaccharides (α-/β-galactosides, α-glucosides, fructooligosaccharides), as well as glucoronate, sorbitol, erythritol, and mannoheptulose. All five newly predicted TRAP systems and three TTT-family transporters are specific to sugar acids (hexuronates, arabinonate, galactonate, fuconate) and hexose diacids (glucarate, galactarate). We also identified 12 novel single-component sugar permeases located in the inner membrane (AraT, BglT, BglT^II^, GalP^II^, FruT, MalP, COG2211), three TonB-dependent outer membrane transporters (with predicted specificities to sucrose and β-glucosides) and two novel outer membrane porins (possibly involved in uptake of glucose and glycerol).

Transcriptional regulation is another highly variable aspect of the sugar utilization pathways in UCC genomes. Indeed, 42 of the 88 TFs tentatively associated this UCC sugar utilization pathways are non-orthologous to their counterparts previously characterized in other bacteria, as captured in the RegPrecise database (Novichkov et al., 2013). We identified candidate TFBSs and reconstructed regulons for 60 TFs including 30 novel regulators (Supplementary Table S2). The majority of genes from sugar catabolic pathways were identified as candidate members of respective sugar-specific TF regulons in UCC genomes.

In *Rhodobacteriaceae* bin08, we identified a new gene locus encoding an ABC-family transporter, an aldolase from the tagatose-1,6-bisphosphate aldolase family (COG3684) and two kinases (COG1940 from the ROK family and COG0529 from the adenylylsulfate kinase family). The substrate-binding component of this ABC transport system has 83% similarity to Avi_5339 from *Agrobacterium vitis*, which was previously found to bind mannoheptulose (Steven Almo and John Gerlt, unpublished observation). Mannoheptulose is a heptose, which is structurally similar to D-tagatose. Based on these observations and known substrate specificities for other kinases from the COG1940 and COG0529 families, we propose the following hypothetical pathway for mannoheptulose utilization. The COG1940 kinase first phosphorylates the substrate to produce mannoheptulose-7-phosphate, then the COG0529 kinase further produces mannoheptulose-1,7-biphosphate, which is subject to the COG3684 aldolase reaction producing glycerone phosphate and erythrose-4-phosphate.

Three UCC members are predicted to utilize xylose though the classical isomerase pathway (XylB, XylA), whereas both *Halomonas* species have a different pathway for xylose utilization including xylose dehydrogenase XylD. In *Caulobacter crescentus*, xylose is converted to α-ketoglutarate by xylose dehydrogenase, xylonolactonase, and xylonate dehydratase (Stephens et al., 2007). However, we have not identified candidate genes for xylonolactonase and xylonate dehydratase in *Halomonas* spp. In contrast, the xylose utilization operon in *Halomonas* spp. encodes two hypothetical enzymes, a sugar phosphate isomerase/epimerase (COG1082) and a Gfo/Idh/MocA-family oxidoreductase (COG0673), however, their exact biochemical functions require further experimental characterization.

Another yet unknown pathway involved in rhamnose utilization was identified in *Rhodobacteriaceae* bin08. Its putative rhamnose catabolic locus contains genes encoding the RhaFGHJ transporter and the RhaM mutarotase, however, bin08 lacks other candidate genes required for utilization of rhamnose (Rodionova et al., 2013b). However, function of other six genes in this locus encoding putative enzymes is unclear. Comparative genomics shows that orthologous loci are present in other *Rhodobacteriaceae* genomes and in some cases they include genes encoding rhamnose dehydrogenase and rhamnonate dehydratase, however bin08 lacks orthologs of these genes, suggesting the existence of a novel yet uncharacterized rhamnose catabolic pathway in the *Rhodobacteriaceae* species.

### Growth Phenotype Testing

We tested four UCC isolates including Halomonas sp. HL-48, HL-93, R. calidilacus HL-91, and Marinobacter sp. HL-58 for growth phenotypes on a panel of various hexoses, pentoses, disaccharides, sugar acids, and sugar alcohols (Supplementary Table S4). For the majority of tested carbohydrates we used the Api^TM^50 CH strip assay. Additionally, we confirmed the selected phenotypes by growing the UCC isolates in defined media using carbohydrates as a single carbon source (see example growth curves provided in Supplementary Figure S1). In accordance with the predicted absence of complete carbohydrate utilization pathways, M. excellens HL-55 did not grow on sugars but it grows on glutamate and lactate (data not shown). We were unable to grow other UCC isolates on defined medium, thus below we report results of the growth tests only for above four UCC organisms.

All four tested organisms demonstrated the ability to grow on two hexoses (glucose, fructose) and three disaccharides (trehalose, maltose, sucrose), as well as on glycerol (**Table 2**). Both Halomonas spp. are able to grow on arabinose, xylose, galactose, arabinitol, mannitol, sorbitol and gluconate, whereas HL-91 grow on N-acetylglucosamine, mannose, and cellobiose (α β-glucoside). In addition, HL-93 has growth phenotypes on fucose, mannose, erythritol, inositol, glucoronate, and galacturonate. With a single exception of the fructose and mannose utilization in HL-91 when we were unable to predict specific catabolic pathways, the measured phenotypes are consistent with the reconstructed catabolic pathways.

**Table 2:** Carbon sources that support growth of four heterotrophic UCC members.

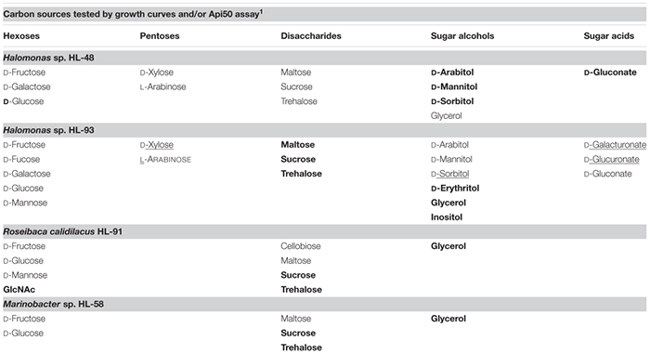

*^1^Carbon sources shown in regular font have positive phenotypes as determined by the Api50 assay. Phenotypes validated by both the Api50 assay and the growth curves measurements are in bold. Phenotypes with measured growth curves but without Api50 tested phenotypes are underlined. GlcNAc, *N-*acetylglucosamine. The detailed comparison of the predicted and experimentally determined growth phenotypes is provided in Supplementary Table S4.*

### Central Carbohydrate Metabolism

**FIGURE 6:**
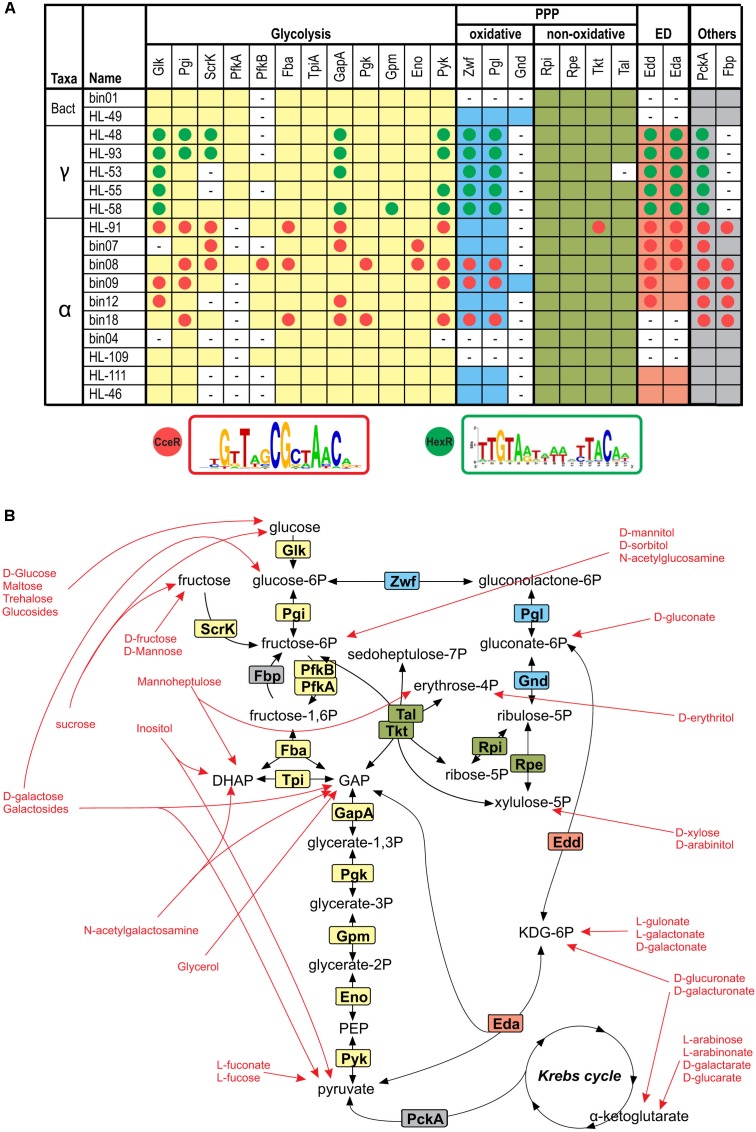
Reconstruction of CCM in heterotrophic UCC organisms. **(A)** Occurrence and regulation of genes involved in glycolysis, oxidative and non-oxidative pentose phosphate (PP) and Entner-Doudoroff (ED) glycolytic pathways in the analyzed genomes. Aliases for analyzed UCC genomes are described in **Table 1**. The presence of genes encoding respective enzymes is shown by background colors matching similar pathways. Candidate members of HexR and CceR regulons are shown by green and red circles, respectively. Sequence logos for the predicted DNA motifs of HexR and CceR regulators are given below the table. The details on the predicted regulator-binding sites and gene locus tags for the identified genes are included in Supplementary Table S2. **(B)** Overview of the CCM and metabolite entrances from peripheral carbohydrate utilization pathways. Enzymes are colored according to the occurrence table in **(A)**. Red arrows point toward final products of each carbohydrate utilization pathway reconstructed in this study.

Peripheral catabolic pathways produce intermediates that are further catabolized through the CCM pathways including the glycolysis, the oxidative and non-oxidative branches of the pentose phosphate (PP) pathway and the Entner-Doudoroff (ED) pathway (**Figure 6**). To understand downstream parts of the reconstructed catabolic pathways, we searched the genomes of 17 heterotrophic UCC members for known CCM genes (Supplementary Table S3). The complete glycolysis pathway was identified in 13 species, whereas the ED pathway (Zwf, Pgl, Edd, Eda) is present in 12 organisms including three α-proteobacteria that have missing 6-phosphofructokinase Pfk. The bin04 genome lacks the Pfk, Glk, Pyk, Zwf, Edd, and Eda enzymes, suggesting this organism cannot utilize carbohydrates. In agreement with these findings, our genomic analysis did not identify any carbohydrate utilization pathway in bin04. The non-oxidative PP pathway, which is essential for nucleic acid synthesis, was found in all studied genomes. The oxidative PP pathway, which is characterized by the presence of Gnd (in addition to Zwf and Pgl), was identified only in HL-49 and bin09. Thus, bin09 has the most diverse set of CCM pathways involved in sugar utilization.

Bacterial CCM genes are often controlled by global transcriptional regulators, such as HexR and FruR in γ-proteobacteria, and CceR and GluR in α-proteobacteria (Ravcheev et al., 2014; Imam et al., 2015) Orthologs of HexR and CceR were identified in UCC proteobacteria, and their regulons were reconstructed using the comparative genomics approach (Supplementary Table S3 and **Figure 6**). The RpiR-family regulator HexR that responds to 2-keto-3-deoxy-gluconate-6P (Leyn et al., 2011) was identified in all five γ-proteobacteria, where it mostly regulates the glycolysis and ED pathway genes, as well as the gluconeogenesis gene *pckA*. The LacI-family regulator, CceR, that senses gluconate-6P (Imam et al., 2015) was identified in six α-proteobacteria from the *Rhodobacteriaceae* family, where it controls a broad range of genes involved in the glycolysis, gluconeogenesis, ED and PPP pathways, as well as the ATP synthase genes. Thus, the transcriptional control of the CCM and peripheral sugar catabolic pathways in heterotrophic UCC organisms is mediated by distinct global and local TFs that co-regulate non-overlapping sets of genes.

## Conclusion

By applying the subsystem-based comparative genomics approach, we reconstructed carbohydrate utilization pathways and predicted catabolic potential for heterotrophic members of UCC consortia. Overall, the reconstructed sugar utilization subsystems include almost 800 genes unevenly distributed across 17 analyzed genomes. Functional roles of 171 genes were first proposed in this study. 13 UCC members were predicted to utilize at least some carbohydrates as a source of carbon and energy using the dedicated catabolic pathways (**Figure 1**). The *Halomonas* strains HL-48 and HL-93 have capabilities to utilize over 20 substrates including pentoses, hexoses, disaccharides, sugar acids, and alcohols. *Rhodobacteriaceae* bin08 is able to grow on 18 substrates including mannoheptulose, a heptose for which we proposed a novel catabolic pathway/transporter/regulon. Each catabolic pathway includes a specialized, often multicomponent, transport system and a set of intracellular enzymes catalyzing biochemical transformations of a particular sugar into one of the common CCM intermediates (**Figure 6**). For the majority of reconstructed pathways we also mapped their cognate TFs that are involved in transcriptional regulation (induction) of catabolic genes, and reconstructed the TF regulons, often allowing the identification of missing transporters and enzymes. Further assessment of two *Halomonas* strains and two other UCC organisms for the growth on a large number of carbon sources allowed us to confirm the majority of the *in silico* predicted catabolic phenotypes. The results of the Api50 testes and extended growth profiling revealed a remarkable consistency between the predicted and observed phenotypes.

**FIGURE 7:**
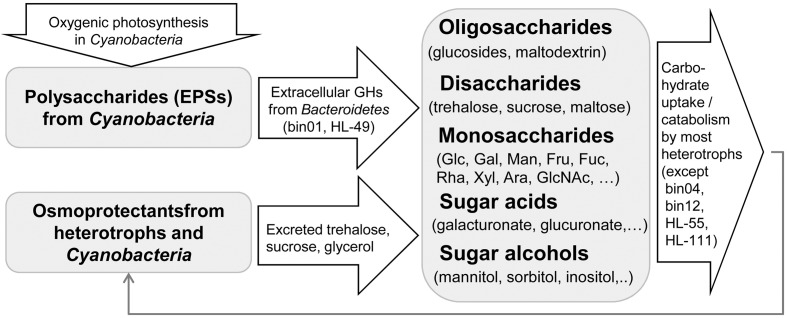
Proposed scheme for carbon flow in the UCC.

Exopolysaccharides produced by autotrophic *Cyanobacteria* serve as the main carbon sources for heterotrophic UCC members (**Figure 7**). Secreted GHs (such as glucosidases) identified in the two *Bacteroidetes* members could benefit the other carbohydrate-utilizing heterotrophs by producing transportable mono- and oligosaccharides. Utilization pathways for disaccharides (maltose/trehalose/sucrose), β-glucosides and glucose are the most abundant among UCC members (present in 8–10 genomes). All other peripheral pathways are present in <30% of the UCC organisms, and among them, 15 pathways are present only in 1–2 genomes. The obtained carbohydrate utilization profiles of UCC heterotrophs are in agreement with the carbohydrate composition of cyanobacterial EPSs (Pereira et al., 2009). Cumulatively, they have pathways to utilize most of the known monosaccharide components of EPSs including glucose, galactose, mannose, fructose, xylose, arabinose, fucose, rhamnose, glucuronate, and galacturonate. Glucosides and disaccharides could be also generated from EPSs. Additionally, trehalose and sucrose are known osmolytes produced by many bacteria to protect them against high salinity levels. There are a plenty of osmoprotectants released into the UCC growth media including glycerol, gluconate, trehalose, and sucrose (Cole et al., 2014). Indeed, the glycerol utilization pathway was identified in five UCC members, while gluconate is utilized by two *Halomonas* isolates. Thus we propose that the UCC community has two levels of carbon donors: (i) *Cyanobacteria* that provide both EPS and osmoprotectants, and (ii) heterotrophic bacteria that could use the cyanobacteria-generated substrates to synthesize their own osmoprotectants and in turn share them with the community. Four UCC members do not rely on carbohydrates for growing. These organisms could use other by-products (such as lactate) secreted by *Cyanobacteria* and other heterotrophs and thus serve as yard cleaners for the community.

Our ability to predict a phenotype of organism from its genome is one of the key goals in microbiology. A systematic application of this omics approach for metabolic reconstruction in a growing number of microbial genomes would allow us to establish the capability of highly accurate automated annotation and assertion of carbohydrate catabolic phenotypes in microbial communities.

## Author Contributions

SL performed the majority of bioinformatics analysis and wrote the paper; YM performed experimental validation of UCC isolate phenotypes; MR annotated the analyzed genomes; DR designed the study, analyzed the obtained data and wrote the paper.

## Conflict of Interest Statement

The authors declare that the research was conducted in the absence of any commercial or financial relationships that could be construed as a potential conflict of interest.

## Footnotes

1 http://www.ebi.ac.uk/ena
