# Supplementary material for: Genomic Reconstruction of Carbohydrate Utilization Capacities in Microbial-Mat Derived Consortia
Source: Front Microbiol. 2017 Jul 13;8:1304. doi: 10.3389/fmicb.2017.01304 (PMC5507952; doi:10.3389/fmicb.2017.01304)
Supplement: Supplementary file 5 [file Image_1.PDF]

Figure S1. Example growth curves for *Marinobacter* sp. HL-58, *Roseibaca calidilacus* HL-91, *Halomonas* sp. HL-48 and *Halomonas* sp. HL-93 grown in defined media supplemented with individual carbohydrates as a single carbon and energy source.

An optical density (OD_{600}) was measured to monitor cell growth during 60 – 100 hours using a plate reader instrument Norden Lab Professional-Bioscreen. 250 µL culture volumes in the 100 well Bioscreen plate, and each growth experiments were performed in 10 replicates.

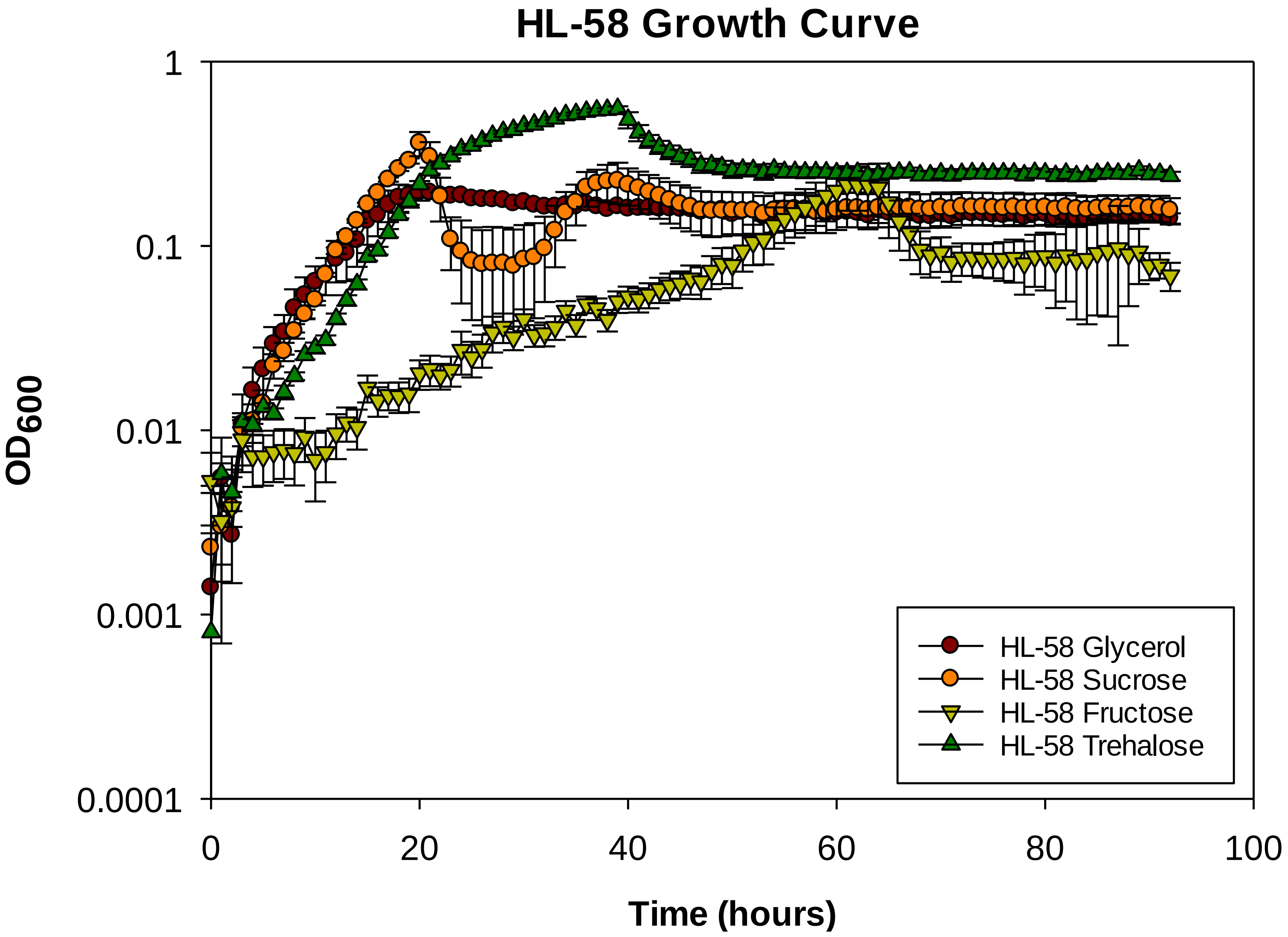
HL-58 Growth Curve
OD600
Time (hours)
1
0.1
0.01
0.001
0.0001
0
20
40
60
80
100
HL-58 Glycerol
HL-58 Sucrose
HL-58 Fructose
HL-58 Trehalose

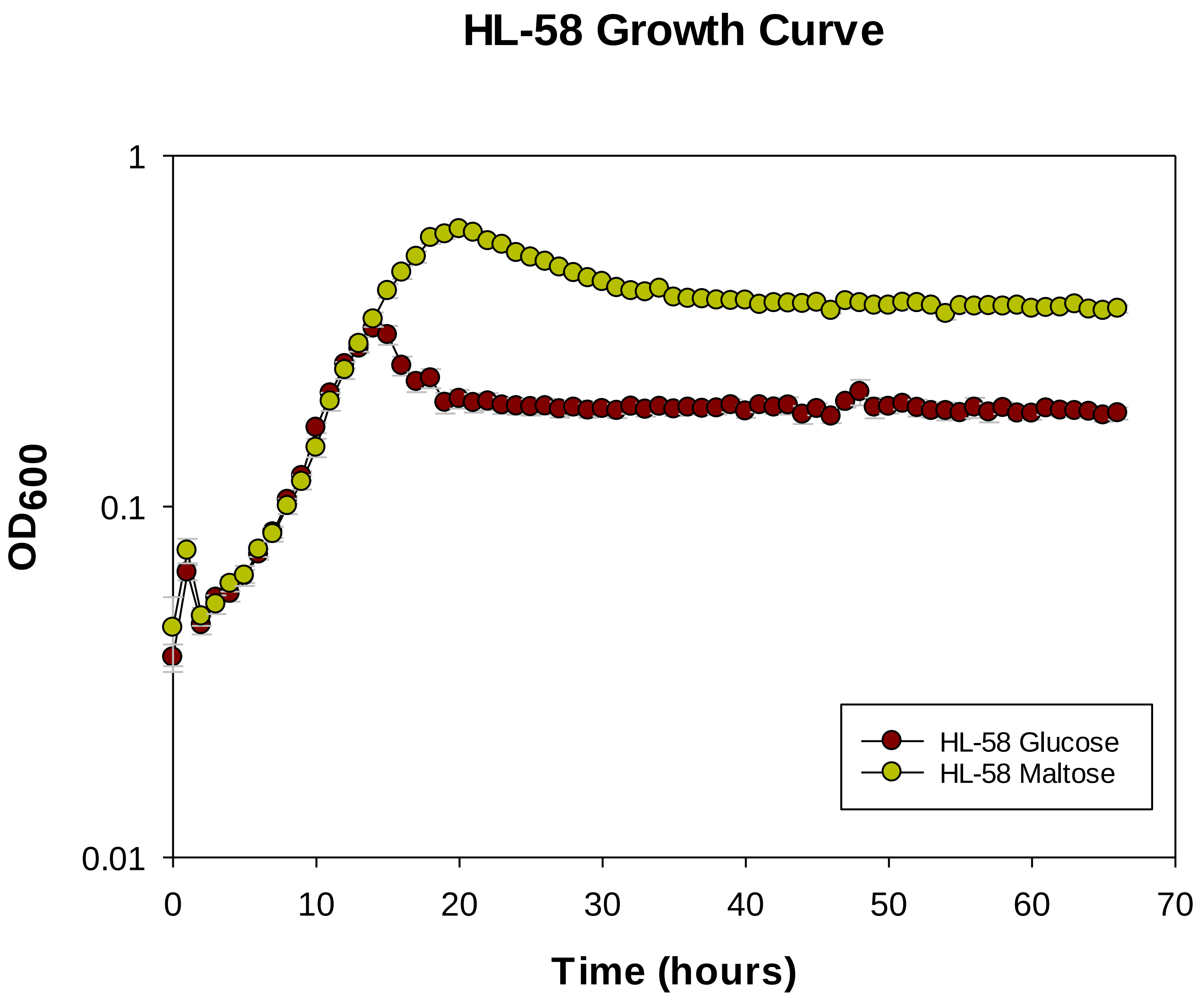
HL-58 Growth Curve
1
0.1
0.01
OD_{600}
0
10
20
30
40
50
60
70
Time (hours)
HL-58 Glucose
HL-58 Maltose

HL-91 Growth Curve

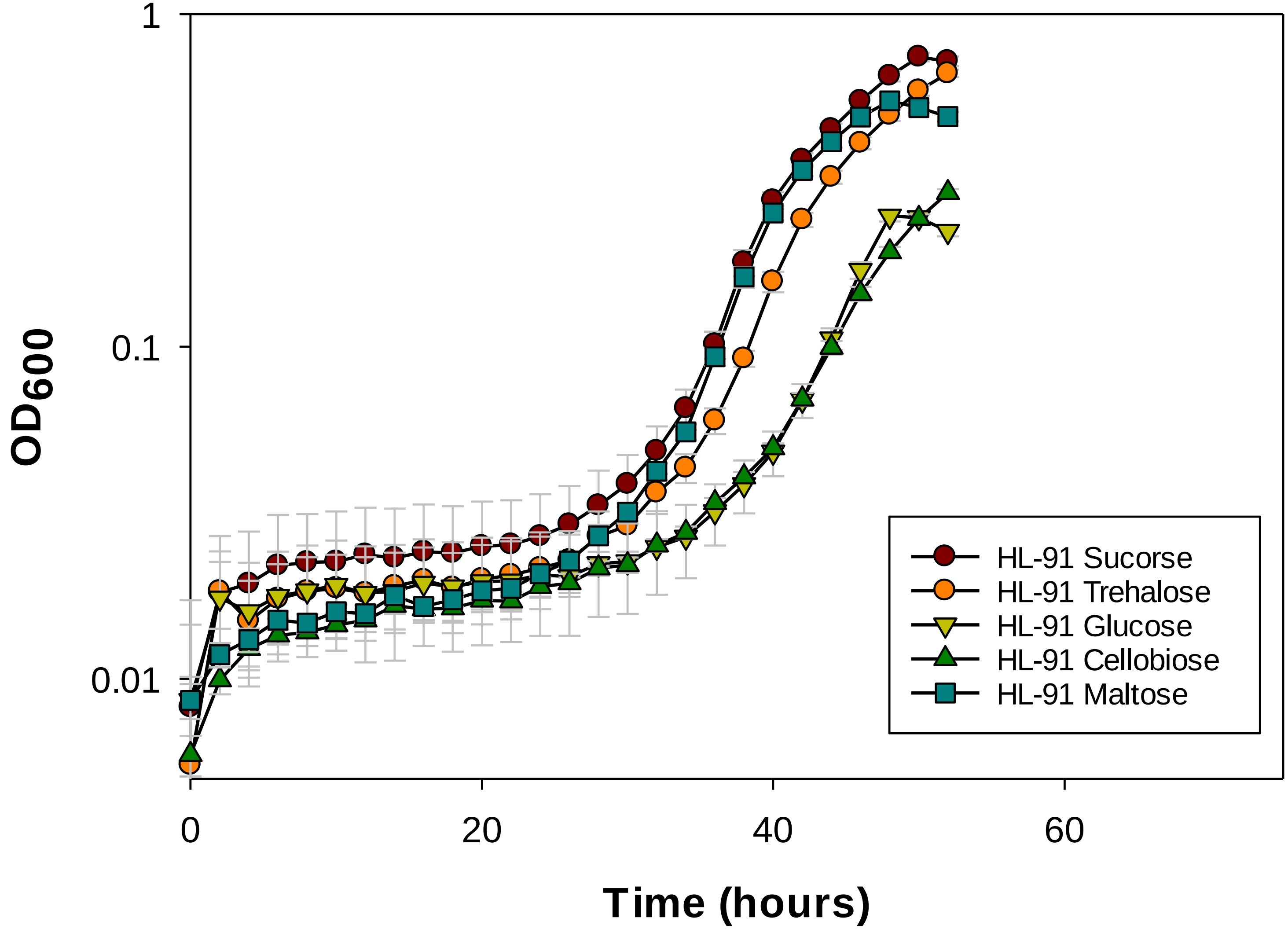

Notes: 5mM each carbon source, experiments were done in 10 reps

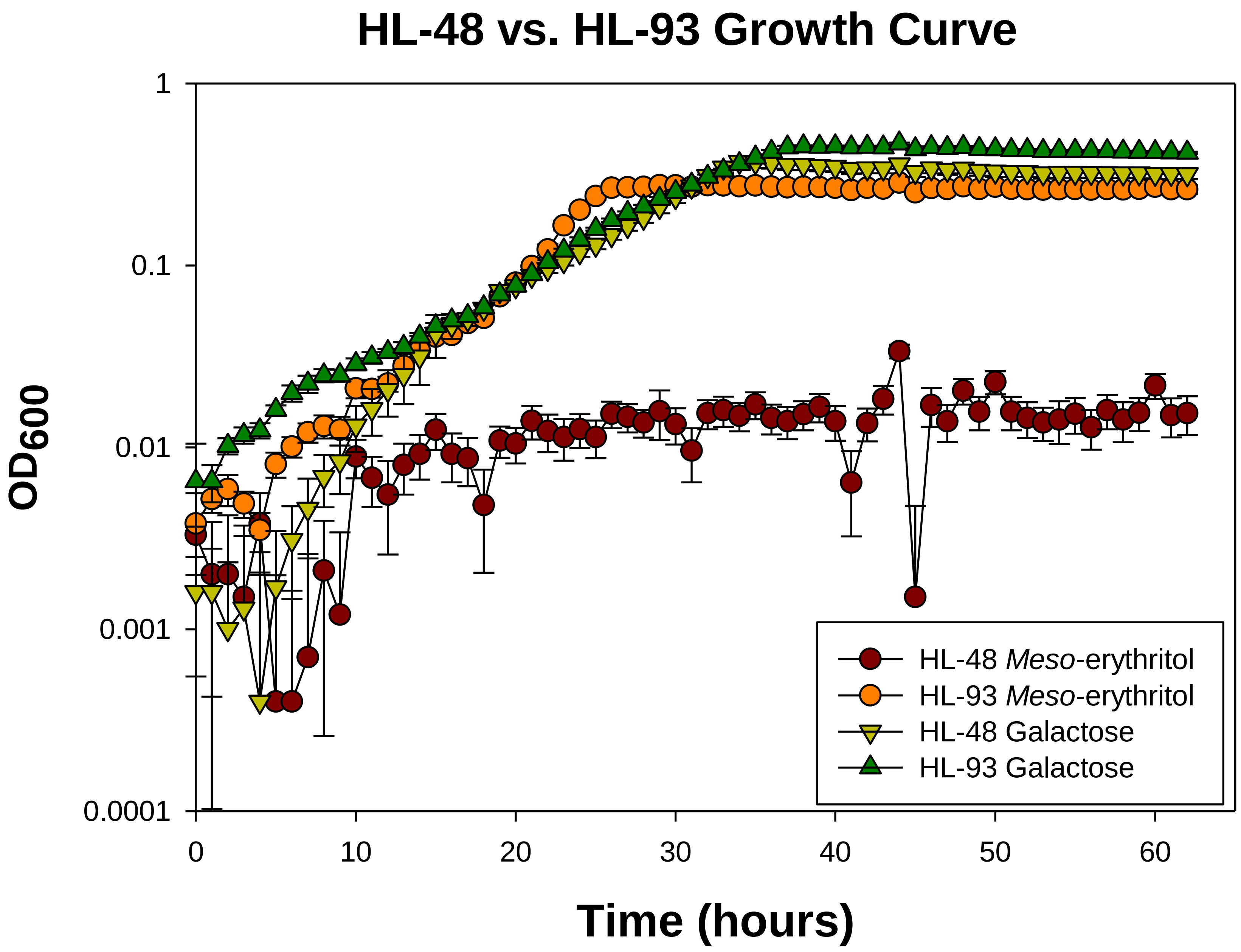
HL-48 vs. HL-93 Growth Curve
OD600
Time (hours)
1
0.1
0.01
0.001
0.0001
0
10
20
30
40
50
60
HL-48 Meso-erythritol
HL-93 Meso-erythritol
HL-48 Galactose
HL-93 Galactose

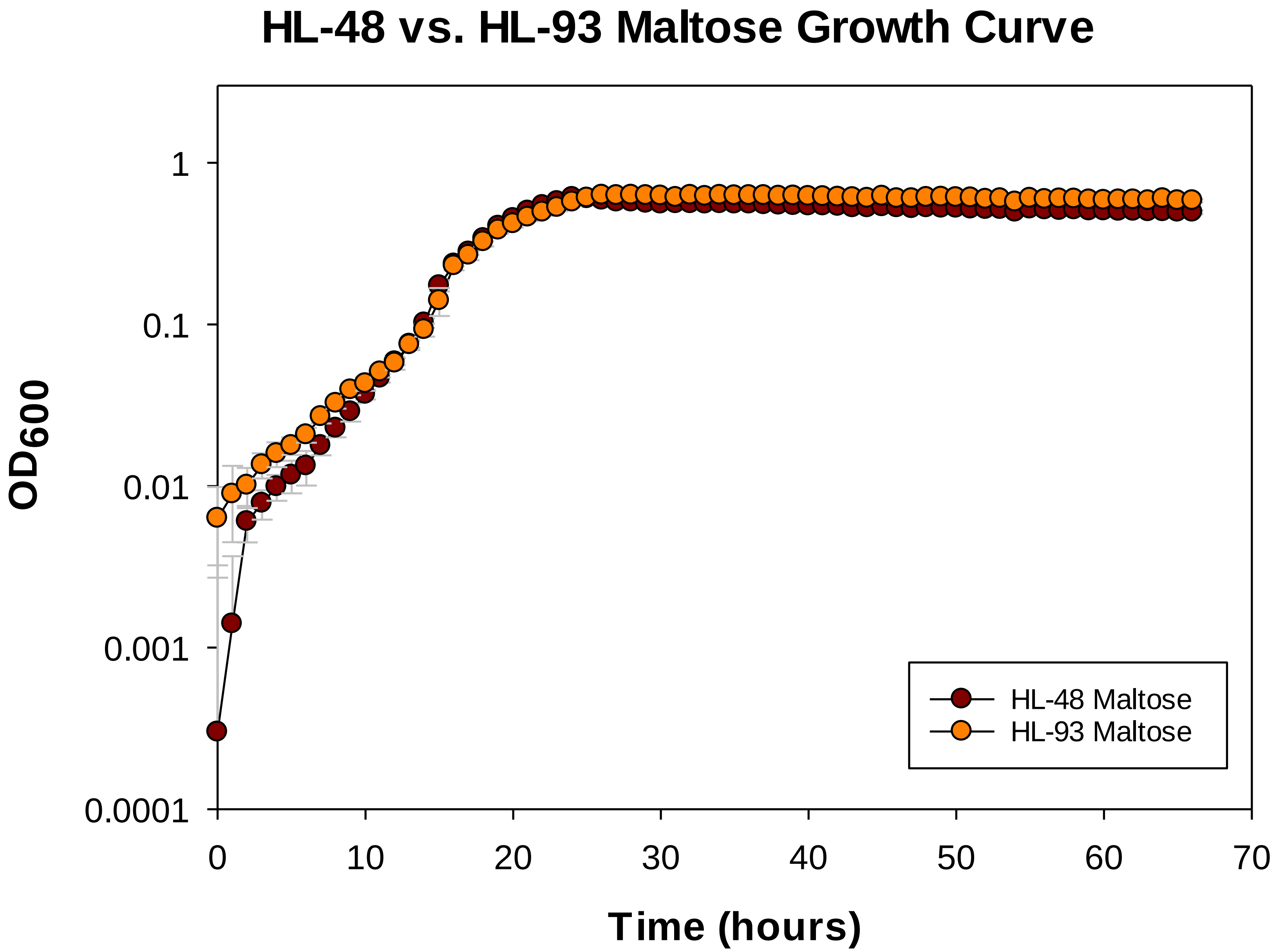
HL-48 vs. HL-93 Maltose Growth Curve
1
0.1
0.01
0.001
0.0001
OD600
0
10
20
30
40
50
60
70
Time (hours)
HL-48 Maltose
HL-93 Maltose

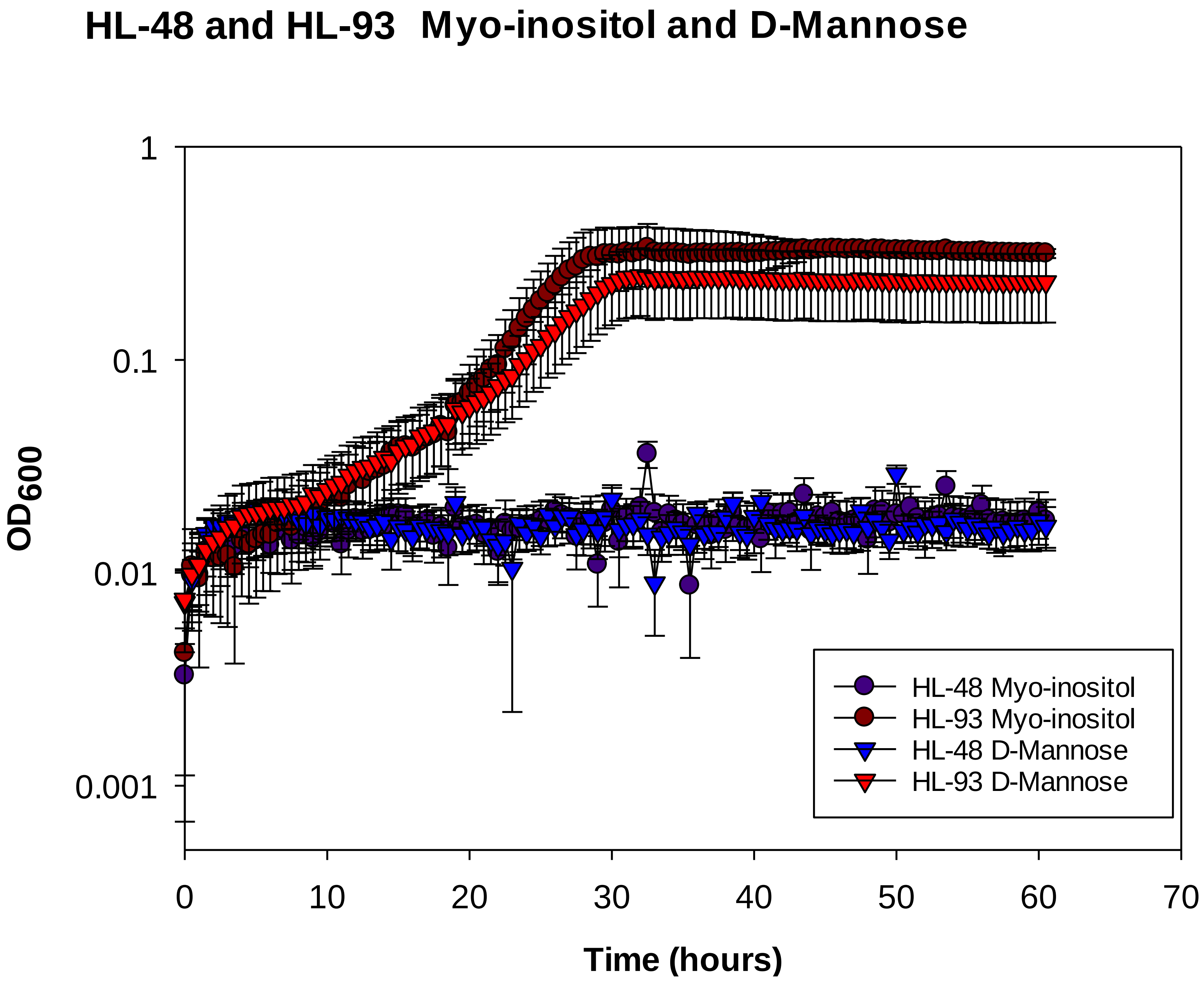
HL-48 and HL-93 Myo-inositol and D-Mannose
OD600
1
0.1
0.01
0.001
0
10
20
30
40
50
60
70
Time (hours)
HL-48 Myo-inositol
HL-93 Myo-inositol
HL-48 D-Mannose
HL-93 D-Mannose

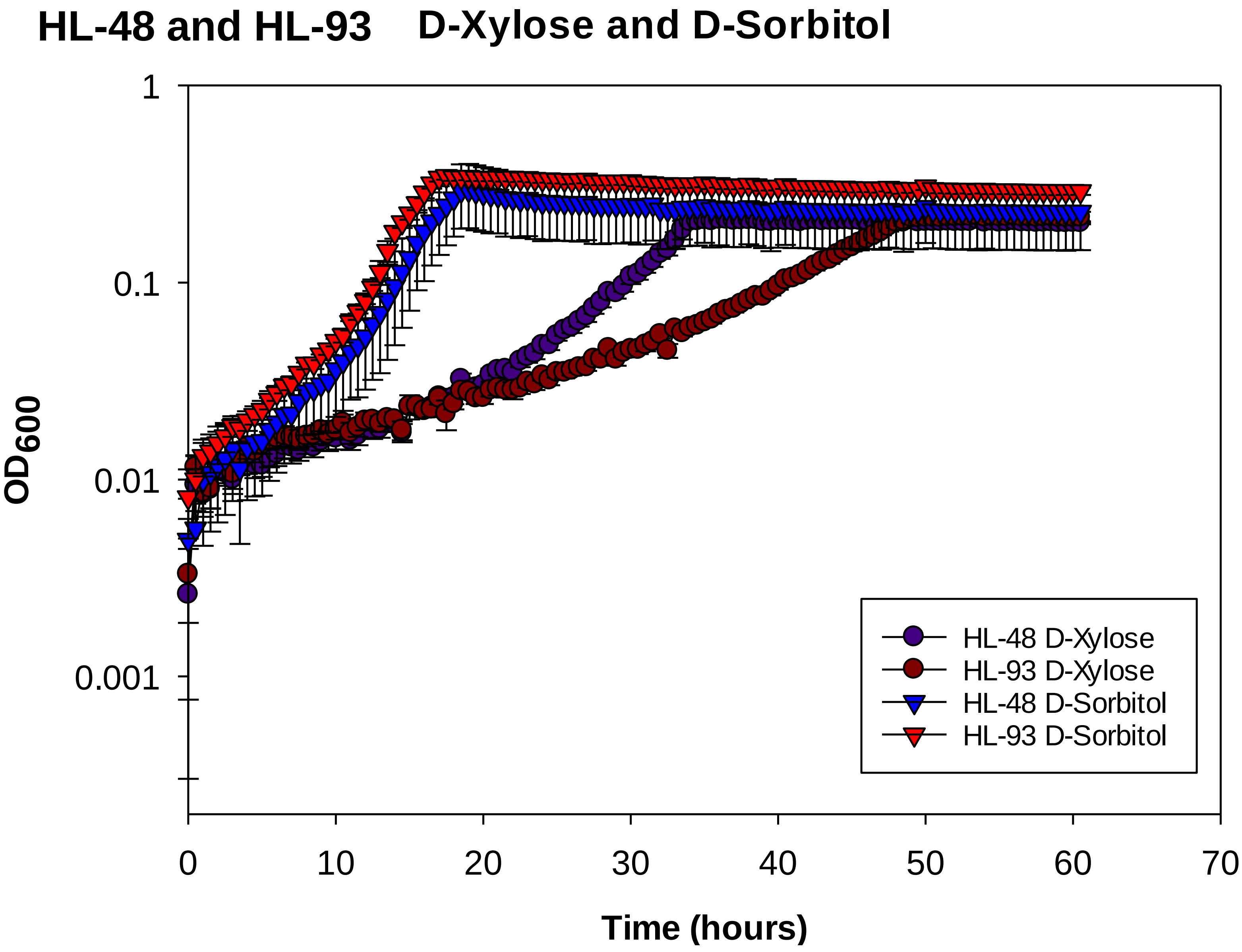

HL-48 and HL-93 D-Xylose and D-Sorbitol
1
0.1
0.01
0.001
OD_{600}
0
10
20
30
40
50
60
70
Time (hours)
HL-48 D-Xylose
HL-93 D-Xylose
HL-48 D-Sorbitol
HL-93 D-Sorbitol

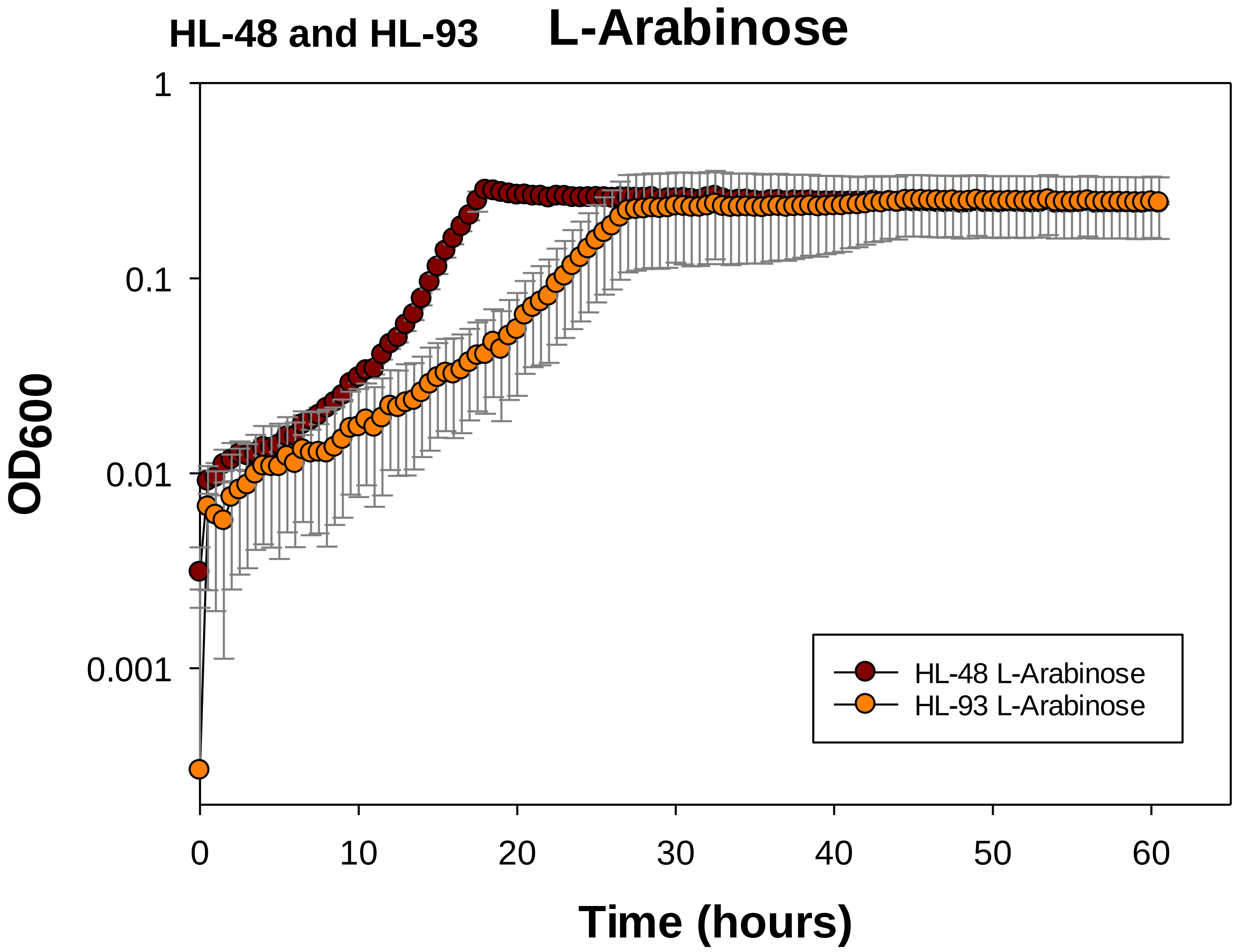
HL-48 and HL-93
L-Arabinose
OD600
1
0.1
0.01
0.001
0
10
20
30
40
50
60
Time (hours)
HL-48 L-Arabinose
HL-93 L-Arabinose

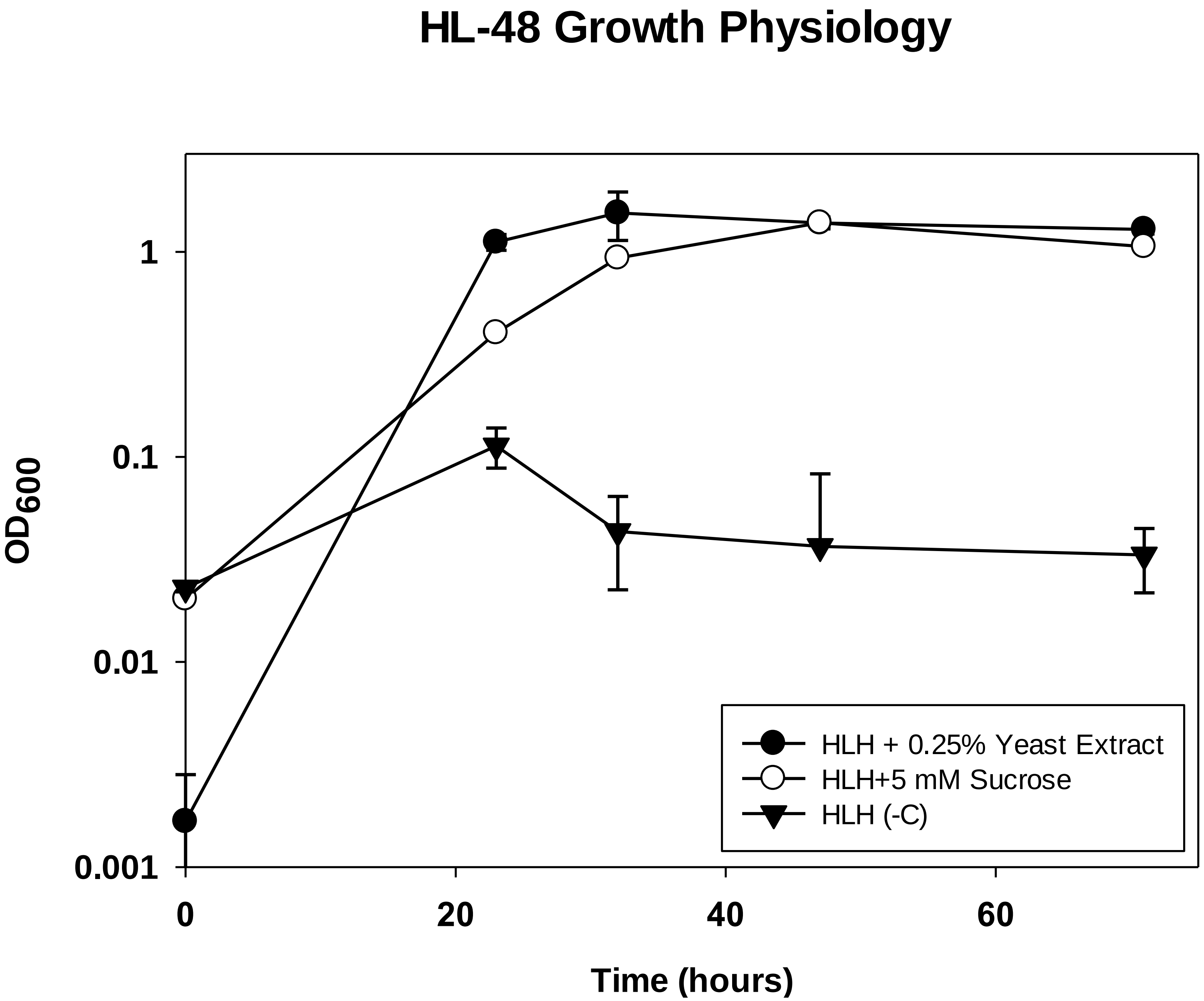
HL-48 Growth Physiology
OD600
1
0.1
0.01
0.001
0
20
40
60
Time (hours)
HLH + 0.25% Yeast Extract
HLH+5 mM Sucrose
HLH (-C)
